# Supplementary material for: Deletion of wfs1 Impairs Oligodendrocyte Precursor Cells Dorsal Distribution and Myelination Through the wfs1-hmgcs1 Axis in Zebrafish
Source: Biology (Basel). 2026 Aug 16;15(16):1402. doi: 10.3390/biology15161402 (PMC13510151; doi:10.3390/biology15161402)
Supplement: Supplementary file 1 [file biology-15-01402-s001.zip › File S1-Transcriptome sequencing data and differentially expressed genes (DEGs) in wfs1-- zebrafish at 5 dpf/1.GO/wfs1KOvsWT/GSEA/wfs1KO/CONDENSED_CHROMOSOME/CONDENSED_CHROMOSOME(GO_0000793).html]

Details for gene set CONDENSED\_CHROMOSOME(GO:0000793)[GSEA]


|  || Dataset | deg.sample |
| Phenotype | sample.cls |
| Upregulated in class | wfs1KO |
| GeneSet | CONDENSED\_CHROMOSOME(GO:0000793) |
| Enrichment Score (ES) | 0.48713404 |
| Normalized Enrichment Score (NES) | 2.0268457 |
| Nominal p-value | 0.0 |
| FDR q-value | 0.045916893 |
| FWER p-Value | 0.164 |
Table: GSEA Results Summary

  

Fig 1: Enrichment plot: CONDENSED\_CHROMOSOME(GO:0000793)
  
Profile of the Running ES Score & Positions of GeneSet Members on the Rank Ordered List

  

| PROBE | DESCRIPTION   (from dataset) | GENE SYMBOL | GENE\_TITLE | RANK IN GENE LIST | RANK METRIC SCORE | RUNNING ES | CORE ENRICHMENT || 1 | 393176 | rad9a |  |  | 53 | 4.876 | 0.1401 | Yes |
| 2 | 492707 | ncapg |  |  | 55 | 4.865 | 0.2815 | Yes |
| 3 | 553132 | syce2 |  |  | 706 | 2.577 | 0.3359 | Yes |
| 4 | 406487 | rad51 |  |  | 855 | 2.308 | 0.3984 | Yes |
| 5 | 559917 | msh4 |  |  | 1297 | 1.810 | 0.4371 | Yes |
| 6 | 557127 | si:dkey-119f1.1 |  |  | 1897 | 1.415 | 0.4594 | Yes |
| 7 | 324475 | smc3 |  |  | 2204 | 1.283 | 0.4871 | Yes |
| 8 | 797304 | smc6 |  |  | 3819 | 0.865 | 0.4616 | No |
| 9 | 564533 | stag1a |  |  | 4778 | 0.708 | 0.4521 | No |
| 10 | 100037337 | ncapg2 |  |  | 5159 | 0.660 | 0.4593 | No |
| 11 | 322275 | rad21a |  |  | 7470 | 0.455 | 0.4000 | No |
| 12 | 393872 | rad1 |  |  | 7755 | 0.439 | 0.4039 | No |
| 13 | 567168 | stag2a |  |  | 7772 | 0.439 | 0.4161 | No |
| 14 | 570062 | ncaph |  |  | 8816 | 0.380 | 0.3944 | No |
| 15 | 394241 | fancd2 |  |  | 8870 | 0.376 | 0.4037 | No |
| 16 | 562678 | ncaph2 |  |  | 10454 | 0.276 | 0.3620 | No |
| 17 | 402797 | sycp1 |  |  | 10486 | 0.274 | 0.3689 | No |
| 18 | 552978 | ncapd2 |  |  | 11044 | 0.242 | 0.3585 | No |
| 19 | 570531 | brd4 |  |  | 11479 | 0.218 | 0.3512 | No |
| 20 | 415214 | psmc3ip |  |  | 11676 | 0.207 | 0.3510 | No |
| 21 | 568897 | mei4 |  |  | 11765 | 0.202 | 0.3542 | No |
| 22 | 563903 | rad9b |  |  | 12924 | 0.144 | 0.3220 | No |
| 23 | 393148 | cdc14b |  |  | 12989 | 0.141 | 0.3241 | No |
| 24 | 553544 | nsmce4a |  |  | 13324 | 0.124 | 0.3172 | No |
| 25 | 797354 | smc1b |  |  | 13508 | 0.115 | 0.3148 | No |
| 26 | 557000 | sycp2 |  |  | 13780 | 0.103 | 0.3092 | No |
| 27 | 564240 | si:dkey-6i22.5 |  |  | 14166 | 0.084 | 0.2996 | No |
| 28 | 566831 | rad21b |  |  | 14452 | 0.072 | 0.2927 | No |
| 29 | 563669 | stag1b |  |  | 15122 | 0.040 | 0.2729 | No |
| 30 | 556493 | ncapd3 |  |  | 15347 | 0.030 | 0.2667 | No |
| 31 | 321452 | smc2 |  |  | 15601 | 0.017 | 0.2593 | No |
| 32 | 568360 | spdl1 |  |  | 16090 | -0.005 | 0.2441 | No |
| 33 | 436824 | nsmce1 |  |  | 16764 | -0.025 | 0.2237 | No |
| 34 | 100332682 | bod1l1 |  |  | 17241 | -0.043 | 0.2100 | No |
| 35 | 403060 | smc1al |  |  | 17273 | -0.044 | 0.2103 | No |
| 36 | 678602 | sycp3 |  |  | 18439 | -0.096 | 0.1765 | No |
| 37 | 436865 | ska1 |  |  | 18504 | -0.099 | 0.1773 | No |
| 38 | 559665 | smc1a |  |  | 18605 | -0.104 | 0.1772 | No |
| 39 | 352907 | tmpoa |  |  | 19592 | -0.148 | 0.1505 | No |
| 40 | 100537961 | si:ch211-269e2.1 |  |  | 19825 | -0.158 | 0.1478 | No |
| 41 | 566749 | smc5 |  |  | 20293 | -0.179 | 0.1384 | No |
| 42 | 553029 | rad50 |  |  | 21957 | -0.265 | 0.0938 | No |
| 43 | 564644 | nsmce2 |  |  | 22547 | -0.298 | 0.0840 | No |
| 44 | 108180085 | LOC108180085 |  |  | 23138 | -0.337 | 0.0753 | No |
| 45 | 393349 | mis12 |  |  | 23287 | -0.346 | 0.0807 | No |
| 46 | 449942 | ska2 |  |  | 24238 | -0.409 | 0.0628 | No |
| 47 | 100126022 | cenpo |  |  | 24285 | -0.413 | 0.0733 | No |
| 48 | 678633 | rec8b |  |  | 25097 | -0.454 | 0.0610 | No |
| 49 | 767743 | bod1 |  |  | 25512 | -0.486 | 0.0621 | No |
| 50 | 571082 | rad21l1 |  |  | 25692 | -0.502 | 0.0711 | No |
| 51 | 562522 | nsl1 |  |  | 25879 | -0.518 | 0.0803 | No |
| 52 | 560751 | stag2b |  |  | 26935 | -0.623 | 0.0653 | No |
| 53 | 570479 | ska3 |  |  | 30174 | -1.325 | 0.0021 | No |
| 54 | 100037341 | zgc:162895 |  |  | 30823 | -1.781 | 0.0335 | No |
Table: GSEA details
[plain text format]

  

Fig 2: CONDENSED\_CHROMOSOME(GO:0000793)
  
Blue-Pink O' Gram in the Space of the Analyzed GeneSet

  

Fig 3: CONDENSED\_CHROMOSOME(GO:0000793): Random ES distribution
  
Gene set null distribution of ES for
 **CONDENSED\_CHROMOSOME(GO:0000793)**
